# Comparative Study of Relative Peripheral Refraction in Children With Different Degrees of Myopia

**DOI:** 10.3389/fmed.2022.800653

**Published:** 2022-03-10

**Authors:** Lu Xiaoli, Zheng Xiangyue, Lian Lihua, Huang Yuting, Lin Chuni, Xia Yujie, Wang Zhao, Yu Xiaoyi

**Affiliations:** ^1^Department of Ophthalmology, The First Affiliated Hospital of Guangzhou University of Chinese Medicine, Guangzhou, China; ^2^First Clinical Medical College, Guangzhou University of Chinese Medicine, Guangzhou, China

**Keywords:** multispectral refractive topography (MRT), relative peripheral refraction, retinal relative diopter (RDV), degrees of myopia, children

## Abstract

**Purpose:**

To investigate the difference in the retinal refraction difference value (RDV) using multispectral refractive topography (MRT).

**Methods:**

Ninety myopic participants, who met the enrolment requirements, were examined with an automatic optometer after mydriasis. According to the value of the spherical equivalent (SE), the participants were divided into Emmetropia group (E, +0.5D < SE < −0.5D), Low Myopia (LM, −0.5D < SE ≤ −3D), and Moderate and high Myopia (MM, −3D < SE ≤ −10D). The ocular biological parameters were detected by optical biometrics (Lenstar 900, Switzerland), including axial length (AL), lens thickness (LT), and keratometry (K1, K2). Furthermore, the MRT was used to measure the retinal RDV at three concentric areas, with 15-degree intervals from fovea into 45 degrees (RDV-15, RDV 15–30, and RDV 30–45), and four sectors, including RDV-S (RDV-Superior), RDV-I (RDV-Inferior), RDV-T (RDV-Temporal), and RDV-N (RDV-Nasal).

**Results:**

In the range of RDV-15, there was a significant difference in the value of RDV-15 between Group E (−0.007 ± 0.148) *vs*. Group LM (−0.212 ± 0.399), and Group E *vs*. Group MM (0.019 ± 0.106) (*P* < 0.05); In the range of RDV 15–30, there was a significant difference in the value of RDV 15–30 between Group E (0.114 ± 0.219) *vs*. Group LM (−0.106 ± 0.332), and Group LM *vs*. Group MM (0.177 ± 0.209; *P* < 0.05); In the range of RDV 30–45, there was a significant difference in the value of RDV 30–45 between Group E (0.366 ± 0.339) *vs*. Group LM (0.461 ± 0.304), and Group E *vs*. Group MM (0.845 ± 0.415; *P* < 0.05); In the RDV-S position, there was a significant difference in the value of RDV-S between Group LM (−0.038 ± 0.636) and Group MM (0.526 ± 0.540) (*P* < 0.05); In the RDV-I position, there was a significant difference in the value of RDV-I between Group E (0.276 ± 0.530) *vs*. Group LM (0.594 ± 0.513), and Group E *vs*. Group MM (0.679 ± 0.589; *P* < 0.05). In the RDV-T position, there was no significant difference in the value of RDV-T among the three groups. In the RDV-N position, there was a significant difference in the value of RDV-N between Group E (0.352 ± 0.623) *vs*. Group LM (0.464 ± 0.724), and Group E vs. Group MM (1.078 ± 0.627; *P* < 0.05). The RDV analysis in all directions among the three groups showed a significant difference between RDV-S and RDV-I in Group LM (*P* < 0.05). Moreover, the correlation analysis showed that SE negatively correlated with AL, RDV 30–45, RDV-S, RDV-I, and RDV-N.

**Conclusions:**

In this study, there was a significant difference in the value of RDV among Group E, Group LM, and Group MM, and the value of RDV in Group MM was the highest on the whole. In the range of RDV 30–45, there was a growing trend with the increase in the degree of myopia among the three groups. Furthermore, the SE negatively correlated with AL, RDV 30–45, RDV-S, RDV-I, and RDV-N.

## Introduction

Myopia is one of the most common eye diseases worldwide (1). It is the sixth most common cause of blindness (2), and is one of the major diseases threatening vision in the WHO 2020 Action Program. Globally, the number of individuals with myopia is approximately 1.45 billion, with the highest incidence rate in Asia (3). The causes behind myopia development are not completely clear; however, growing evidence suggests that peripheral retinal refractive status may be closely related to development of myopia (4–6). The peripheral retina of emmetropia has a mild relative myopic refractive state, while the peripheral retina of uncorrected hyperopia has a slightly higher relative myopic refractive status. Moreover, the peripheral retina of an uncorrected myopic eye presents mild relative hyperopia (7).

Multispectral refractive topography (MRT) is a new instrument that uses multispectral imaging technology (MSI). The MRT applies an optical imaging refractive compensation to measure the refractive state of the retina. Recently, this technology has been used to diagnose several diseases through a considerable number of spectral bands and with a great spatial resolution (8, 9). Through a computer depth calculation, the multispectral images captured by the lens can be compared and analyzed, and the actual refractive values of each pixel can be used to draw the corresponding topographic map. The MRT can detect each part of retinal refractive values within 45 degrees with a low measurement error.

This study compared how much the peripheral refraction of the eye differs from the refraction of fovea at three concentric areas (RDV-15, RDV 15–30, and RDV 30–45), and four sectors, including RDV-S, RDV-I, RDV-T, and RDV-N among E, LM, and MM groups. We aimed to evaluate the peripheral retinal RDV differences in different degrees of myopia and to explore the correlation between SE and RDV.

## Data and Methods

### General Data

Ninety myopic children (right eye, 90 eyes in total) participated in this study, including 45 male and 45 female, aged 5–18 years (mean age 10.88 ± 2.95 years). The enrollment of participants in each group is shown in Table 1.

**Table 1:** Enrollment of participants in each group.

**Parameter**	**Group E**	**Group LM**	**Group MM**
Gender (M: F)	14:16	15:15	16:14
Age (Years)	11.3 ± 3.88	10 ± 1.60	12 ± 2.88
DS (D)	−0.04 ± 0.35	−1.28 ± 0.56	−4.38 ± 0.82
DC (D)	−0.28 ± 0.41	−0.42 ± 0.49	−0.68 ± 0.48
SE (D)	−0.18 ± 0.34	−1.48 ± 0.55	−4.13 ± 0.82
K1 (D)	42.68 ± 1.27	42.65 ± 1.15	43.04 ± 1.40
K2 (D)	43.57 ± 1.33	43.71 ± 1.25	44.38 ± 1.49

*DS, Diopter sphere; DC, Diopter cylinder; SE, Spherical equivalent; K, Keratometry*.

### Research Equipment

**Figure 1:**
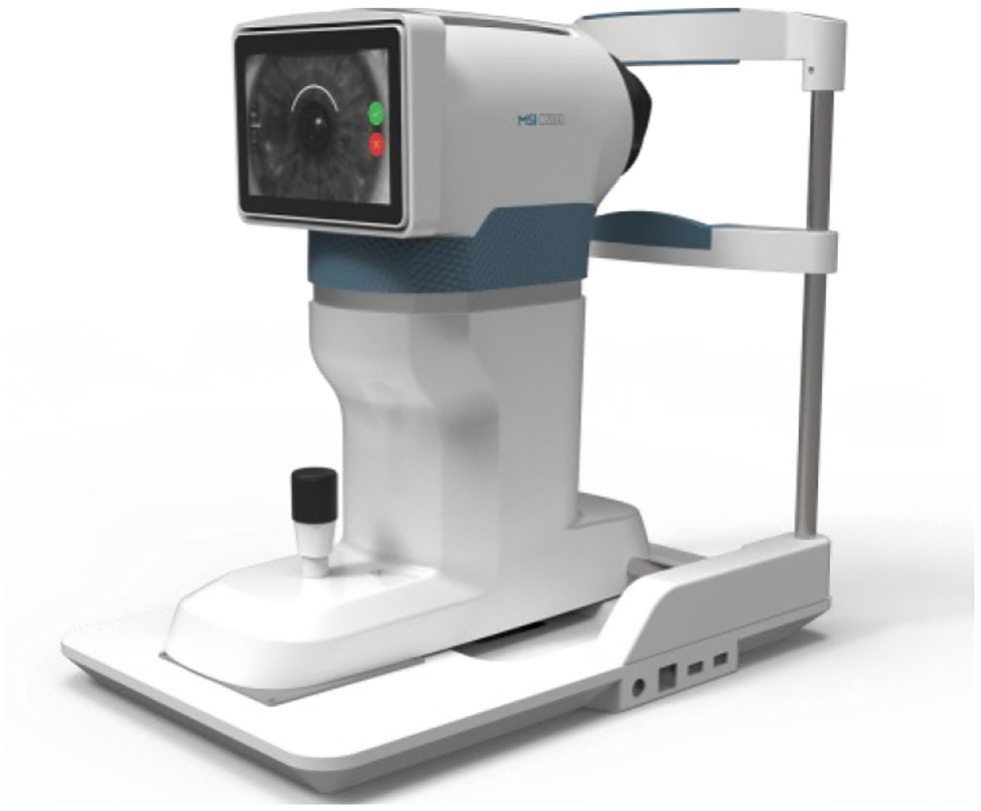
Multispectral refractive topographic instrument. (THONDER, China).

Multispectral fundus camera (Figure 1) (MSI C2000, THONDAR, China); Automatic optometer (Tianle TCS-860, China); and Optical biometrics (Lenstar 900, Switzerland).

### Inclusion and Exclusion Criteria

Inclusion criteria. The subjects are aged 5–18 years and had refractive errors ranging from +0.5D to −10D, astigmatism of less than −2.50D; best-corrected visual acuity of at least 16/20, and no other ocular disease. Exclusion criteria. The strabismus or other visual dysfunction was excluded, as well as major diseases of the eye or general body.

### Methods

**Figure 2:**
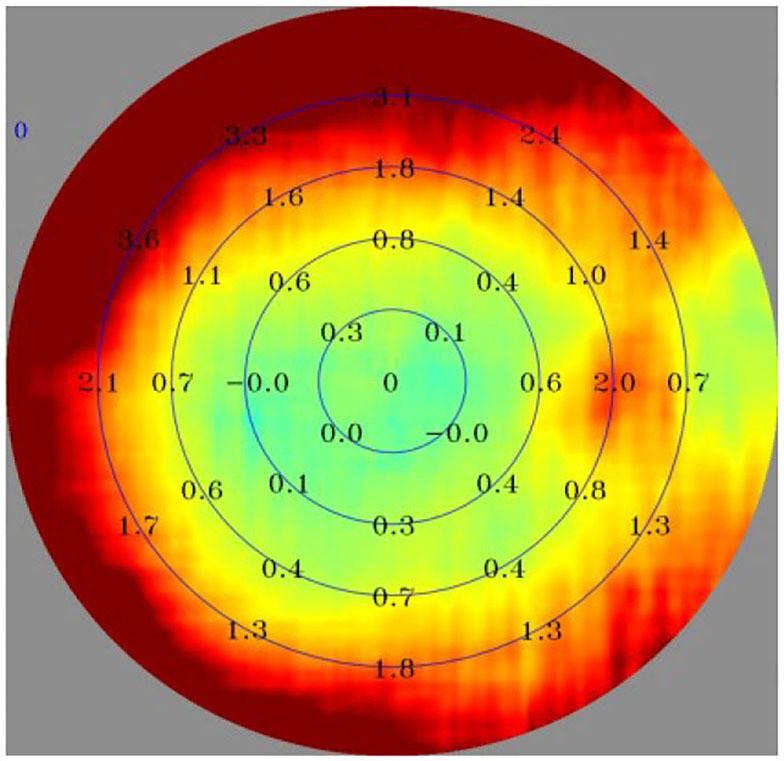
Result of MRT: The innermost circle stands for RDV-10; The second circle stands for RDV-20; The third circle stands for RDV-30; The fourth circle stands for RDV-40.

**Figure 3:**
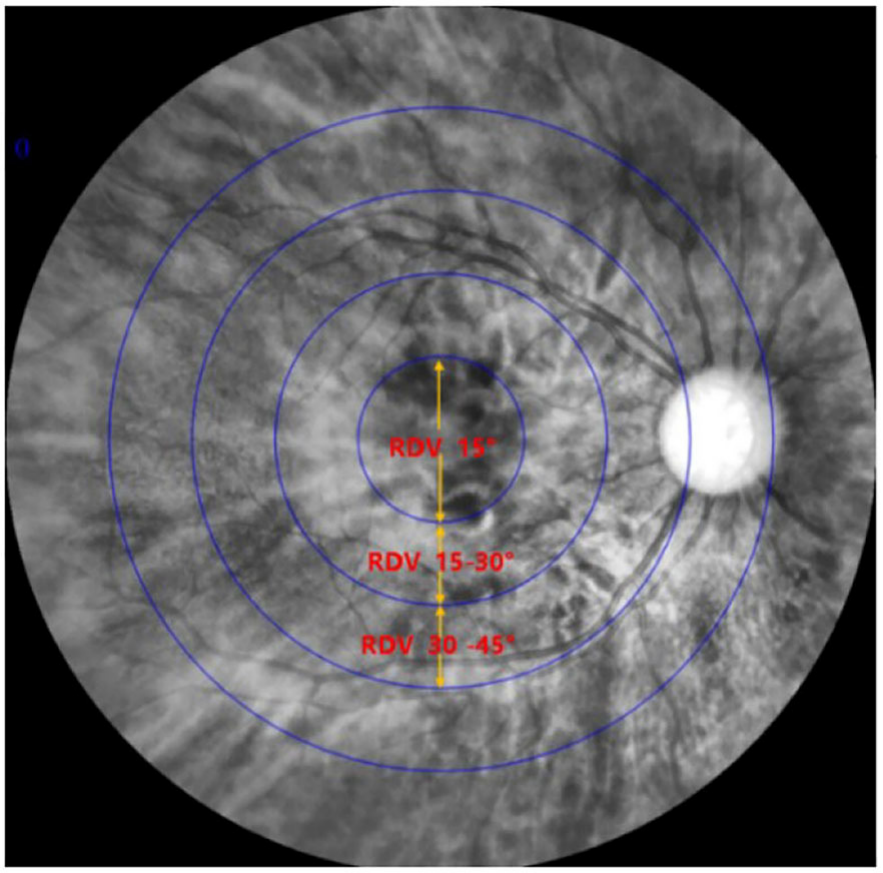
Sketch map of ranges to measure peripheral refraction (RDV-15, RDV 15–30, and RDV 30–45).

**Figure 4:**
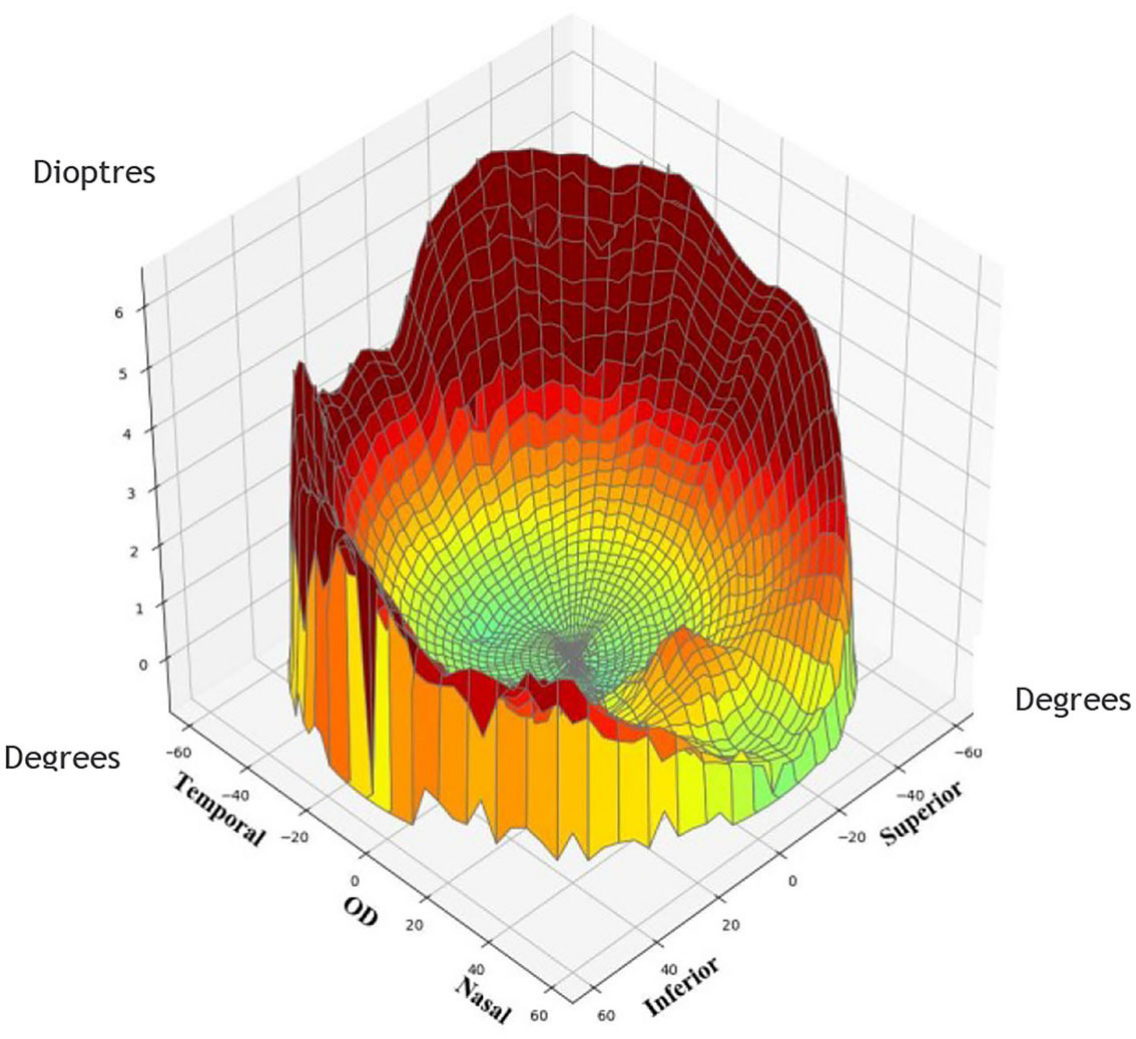
Sketch map of a right eye showing differences between central and peripheric refraction (RDV-S, RDV-I, RDV-T, and RDV-N).

Ninety subjects were measured with MRT, optical biological measuring instrument, and automatic refractometer. The central spherical equivalent (SE) was used to classify the eyes as Emmetropia (E, −0.5 to +0.5D), Low Myopia (LM, −3D to −0.5D), and Moderate and High Myopia (MM, −10D to −3D). After mydriasis, all participants were subjected to optical biometry to measure the ocular biological parameters. Furthermore, the ocular refraction was detected by automatic optometry. The retinal RDV was detected by MRT (Figure 2). The ranges included RDV-15, RDV 30–15, RDV 45–30, RDV-S (RDV-Superior), RDV-I (RDV-Inferior), RDV-T (RDV-Temporal), and RDV -N (RDV-Nasal) (Figures 3, 4). Only the data from the right eye was considered for analysis. The results of refractive measurement were DS/DC × θ (DS = Diopter sphere, DC = Diopter cylinder, and θ = Astigmatism axis). The SE was calculated using the formula DS + DC/2.

### Statistical Analysis

One-way ANOVA was used to analyze the differences of ocular biological parameters and retinal relative peripheral refraction for all the ranges among the three groups. Furthermore, a paired *t*-test was used to compare the nasal and temporal peripheral refraction for each group. The correlation between the SE and the ocular biological parameters and the retinal relative refraction for the different ranges was analyzed by Pearson correlation analysis. *P* < 0.05 was considered statistically significant.

## Results

**Figure 5:**
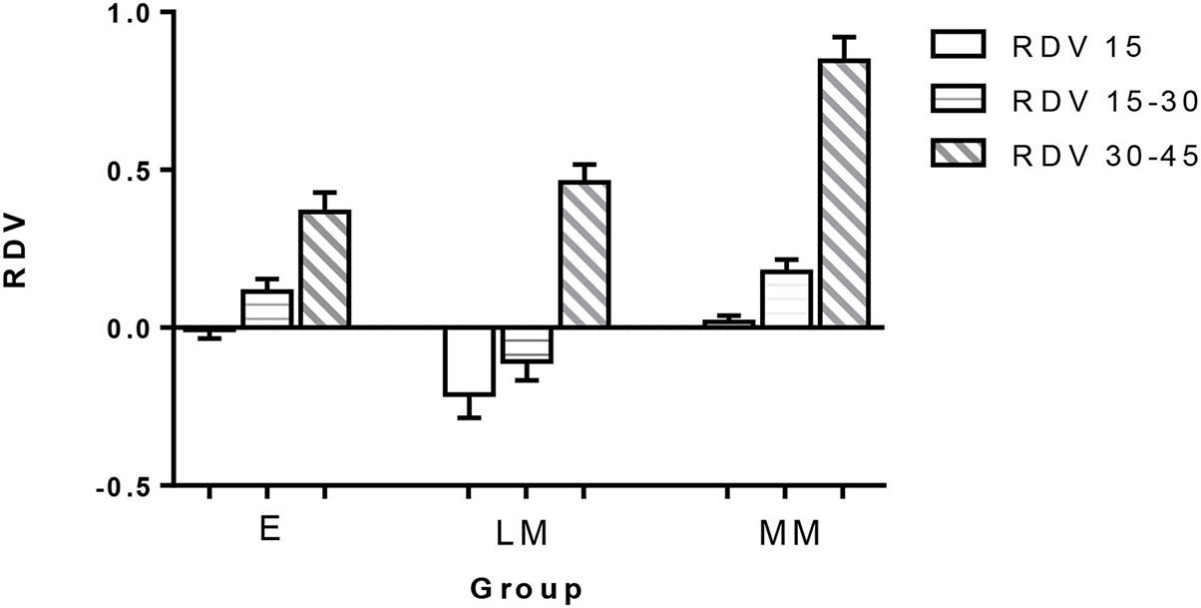
The retinal relative diopter (RDV) values of different peripheral retinal ranges in three groups. In the range of RDV-15, the differences between groups E *vs*. LM and groups E *vs*. MM were statistically significant. In the range of RDV 15–30, the differences between groups E *vs*. LM and groups LM *vs*. MM were statistically significant. In the range of RDV 30–45, the difference between group E vs. LM and group E vs. MM was statistically significant (*P* < 0.05).

**Figure 6:**
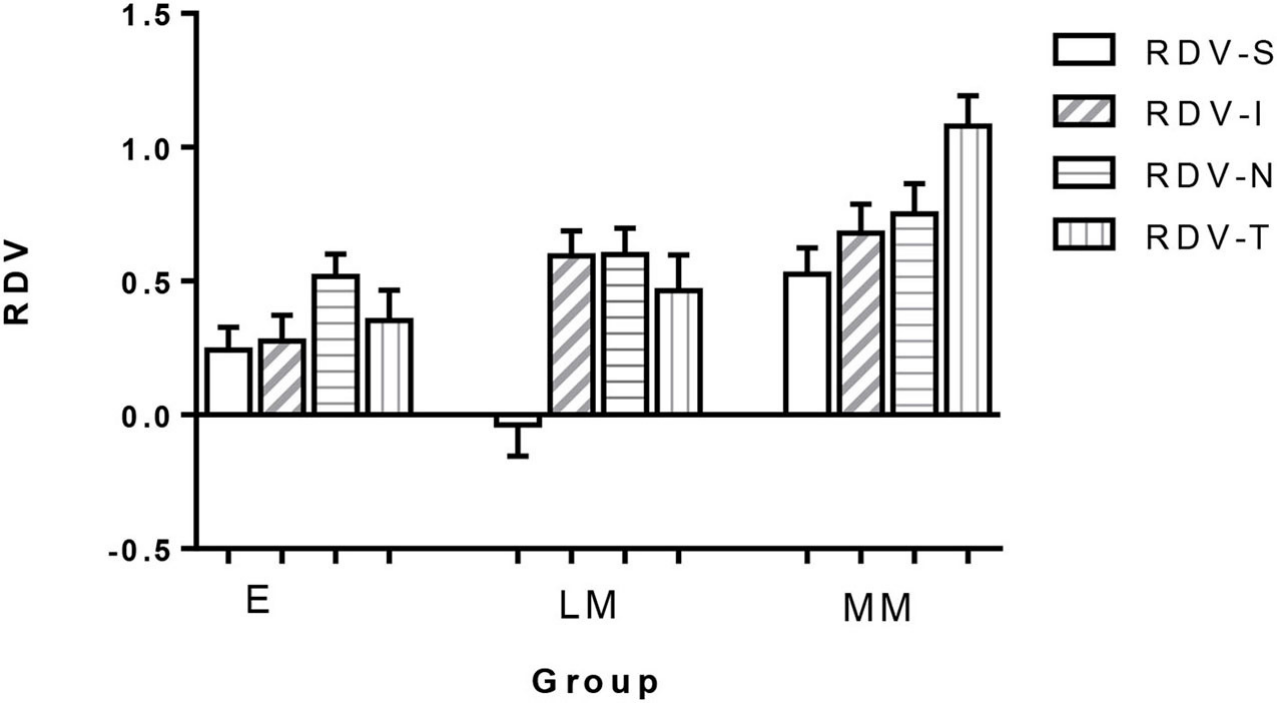
The RDV values [RDV-superior (RDV-S), RDV-inferior (RDV-I), RDV-temporal (RDV-T), and RDV-nasal (RDV-N)] in the peripheral retina of the three groups. In the RDV-S position, there was a significant difference in the value of RDV-S between Group LM and Group MM (*P* < 0.05); In the RDV-I position, there was a significant difference in the value of RDV-I between Group E *vs*. Group LM, and Group E *vs*. Group MM (*P* < 0.05). In RDV-T position, there was no significant difference in the value of RDV-T among the three groups. In the RDV-N position, there was a significant difference in the value of RDV-N. between Group E vs. Group LM and Group E vs. Group MM (*P* < 0.05).

The RDV values of the three groups are shown in Table 2, and their comparisons are illustrated in Figures 5, 6. In the range of RDV-15, there was a significant difference in the value of RDV-15 between Group E (−0.007 ± 0.148) *vs*. Group LM (−0.212 ± 0.399), and Group E *vs*. Group MM (0.019 ± 0.106; *P* < 0.05; Figure 5); In the range of RDV 15–30, there was a significant difference in the value of RDV 15–30 between Group E (0.114 ± 0.219) *vs*. Group LM (−0.106 ± 0.332), and Group LM *vs*. Group MM (0.177 ± 0.209; *P* < 0.05); In the range of RDV 30–45, there was a significant difference in the value of RDV 30–45 between Group E (0.366 ± 0.339) *vs*. Group LM (0.461 ± 0.304), and Group E *vs*. Group MM (0.845 ± 0.415; *P* < 0.05); In the RDV-S position, there was a significant difference in the value of RDV-S between Group LM (−0.038 ± 0.636) and Group MM (0.526 ± 0.540; *P* < 0.05; Figure 6); In RDV-I position, there was significant difference in the value of RDV-I between Group E (0.276 ± 0.530) *vs*. Group LM (0.594 ± 0.513), and Group E *vs*. Group MM (0.679 ± 0.589; *P* < 0.05); In the RDV-T position, there was no significant difference in the value of RDV-T among the three groups. In the RDV-N position, there was a significant difference in the value of RDV-N between Group E (0.352 ± 0.632) *vs*. Group LM (0.464 ± 0.724), and Group E *vs*. Group MM (1.078 ± 0.627; *P* < 0.05).

**Table 2:** The retinal relative diopter (RDV) values in different ranges of the three groups.

	**Group**	**Mean**	**SD**
RDV-15	E	−0.007	0.148
	LM	−0.212	0.399
	MM	0.019	0.106
RDV-15–30	E	0.114	0.219
	LM	−0.106	0.332
	MM	0.177	0.209
RDV-30–45	E	0.366	0.339
	LM	0.461	0.304
	MM	0.845	0.415
RDV-S	E	0.243	0.463
	LM	−0.038	0.636
	MM	0.526	0.540
RDV-I	E	0.276	0.530
	LM	0.594	0.513
	MM	0.679	0.589
RDV-T	E	0.518	0.454
	LM	0.599	0.540
	MM	0.750	0.622
RDV-N	E	0.352	0.623
	LM	0.464	0.724
	MM	1.078	0.627

*RDV-S, RDV-Superior; RDV-I, RDV-Inferior, RDV-T,RDV-Temporal; and RDV-N, RDV-Nasal*.

Upon comparison of RDV-S, RDV-I, RDV-T, and RDV-N in each group using the paired *t*-test, we observed that the difference between RDV-S and RDV-I was statistically significant in Group LM (*P* < 0.05; Table 3). However, there were no statistically significant differences between the RDVs in the other groups.

**Table 3:** Comparative analysis of RDV (RDV-S, RDV-I, RDV-T, and RDV-N) of each group.

			**Mean**	**SD**	***P*-value**
E	Pair 1	RDV-S–RDV-I	−0.029	0.620	0.799
	Pair 2	RDV-T–RDV-N	0.211	0.748	0.133
LM	Pair 1	RDV-S–RDV-I	−0.577	0.894	0.001
	Pair 2	RDV-T–RDV-N	0.129	1.122	0.533
MM	Pair 1	RDV-S–RDV-I	−0.197	0.757	0.165
	Pair 2	RDV-T–RDV-N	−0.306	0.883	0.068

**Figure 7:**
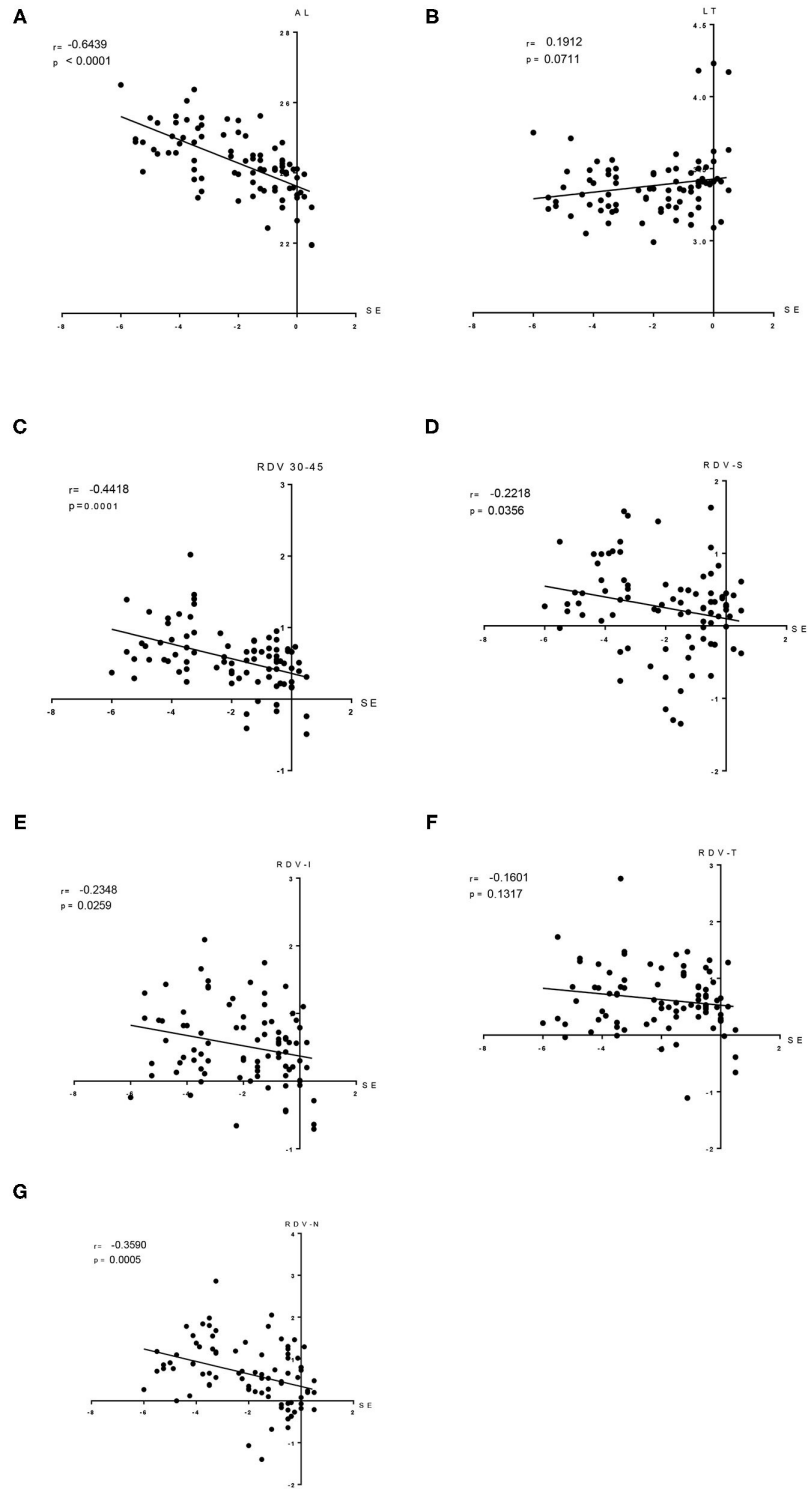
Correlation analysis results. The SE negatively correlated with AL **(A)**; the SE was positively correlated with LT **(B)**; the SE was negatively correlated with RDV 30–45 **(C)**; the SE was negatively correlated with RDV-S **(D)**; the SE was negatively correlation with RDV-I **(E)**; the SE was negatively correlated with RDV-T **(F)**; and the SE was negatively correlation with RDV-N **(G)**. SE, spherical equivalent; and AL, Axial length.

Furthermore, the Pearson correlation analysis indicated a negative correlation of SE with AL (Axial length) (*r* = −0.6439, *P* < 0.05), RDV 30–45 (*r* = −0.4418, *P* < 0.05), RDV-S (*r* = −0.2218, *P* < 0.05), RDV- I (*r* = −0.2348, *P* < 0.05), RDV-N (*r* = −0.3590, *P* < 0.05; Figure 7), and RDV-T (*r* = −0.160, *P* = 0.132). Besides, it indicated a positive correlation of SE with LT (*r* = 0.191, *P* = 0.071).

## Discussion

Myopia is the most common refractive disorder. Several studies have found that peripheral defocus has an important impact on eye growth. Eyes with emmetropia and hyperopia often have relative myopia peripheral defocus, while the eyes with myopia have relative hyperopia peripheral defocus (5, 10).

In recent years, the study of peripheral retinal defocus has gained increased attention. Defocus of the peripheral retina affects the eye length and visual progress in both animals and humans (11–14). Mutti et al. (15) conducted a longitudinal study on 822 cases of children aged 5–14, and discovered that children with myopia had more relative hyperopic defocus than children with emmetropia. However, the operational steps in previous studies on peripheral retinal defocus were relatively complex, which caused measurement errors due to changes in retinal morphology (5). Hence, the advent of MRT made it possible to easily measure the retinal refraction difference value.

This study demonstrated that the degree of myopia increased with the increase in the value of RDV 30–45, and the difference in this value between Group E *vs*. Group LM and Group LM *vs*. Group MM were statistically significant (*P* < 0.05); and the value of RDV in Group MM was the highest on the whole. In a study with 2,286 children with myopia, Allon et al. (16) found that the spherical equivalent positively correlated with myopia-related peripheral retinal changes, which was consistent with our study.

When comparing the horizontal and vertical retinal relative peripheral refraction (RDV-S and RDV-I, respectively) in the Group LM, we found that the difference between RDV-S and RDV-I was statistically significant. Correlation analysis indicated that there was a negative correlation between RDV-T and RDV-N (*r* = −0.5769, *P* = 0.0008) in Group LM. However, there was no significant correlation between the other groups. Hence, we speculated that there may be an imbalance between vertical and horizontal eye development during the development of myopia. Interestingly, Atchison et al. (17) investigated 116 subjects aged 18–35 and discovered that myopia had a greater impact on peripheral refraction along the horizontal rather than vertical field. Through the application of computer digital processing of magnetic resonance images of eyes, Atchison et al. (18) found that with the increase of myopia, the size of all ellipsoids increased with age. Furthermore, they observed that the axial size was larger than the vertical size, which in turn increased the over horizontal size. In the development of myopia, there may be a complex regulatory relationship between the degree of myopia and the ocular growth pattern, which needs to be further investigated.

In this study, the results showed that SE negatively correlated with AL, which meant that with the increase of myopic degree, the axial size would increase correspondingly. However, SE positively correlated with LT (Lens Thickness), which meant that the lens became thinner with the increase of myopia. Mutti et al. discovered that thinner lenses were associated with more hyperopic relative peripheral refractions (15, 19). Smith et al. (6) suggested that peripheral hyperopia was a stimulus for axial prolongation, thus, a corrective treatment should be considered to prescribe lenses to correct not only the central refractive error, but also the peripheral hyperopia defocus.

In recent years, the orthokeratology lens has been widely used in ophthalmology. Orthokeratology can delay the development of myopia by reducing the peripheral hyperopia defocus (20, 21). Some studies have demonstrated that wearing progressive multi-focus soft contact lenses can correct not only the central refractive error but also the peripheral refractive error, thus, delaying the progression of myopia in adolescents (22–25). The MRT, which measures the refractive state of the retina, can be used for forecasting the occurrence and the development of myopia. Furthermore, various kinds of prevention and control methods of myopia are effective, and orthokeratology lens, multiple focal contact lens fitting, excimer laser surgery, and other refractive therapeutic interventions will help solve the problem of myopia in clinical settings.

This study assessed that the peripheral refraction of the eye differs from different retinal eccentricities areas. The study also showed that there was a growing trend with the increase of the degree of myopia in the range of RDV 30–45. Furthermore, the degrees of myopia correlated with AL, RDV 30–45, RDV-S, RDV-I, and RDV-N. Therefore, the peripheral refraction of RDV 30–45 may be closely related to the development of myopia. However, this study has a limitation with respect to the sample size and distribution of the study subjects, since the subjects mainly came from the nearby surrounding areas of our hospital. Hence, a study with a longer period and a larger sample size need to be conducted in the future.

In conclusion, the MRT has a good prospect for clinical application and can detect the relative refraction of the retina, and thereby evaluate the occurrence and development of myopia, intuitively and accurately. Besides, the MRT can guide the clinical treatment of refraction by detecting RDV, which is convenient to guide the selection of a proper treatment plan.

## Data Availability Statement

The raw data supporting the conclusions of this article will be made available by the authors, without undue reservation.

## Ethics Statement

This study has been approved by the Medical Ethics Committee of Guangzhou University of Chinese Medicine (Approval No, K[2020]150). Written informed consent to participate in this study was provided by the participants' legal guardian/next of kin. Written informed consent was obtained from the individual(s), and minor(s)' legal guardian/next of kin, for the publication of any potentially identifiable images or data included in this article.

## Author Contributions

LX and ZX conducted the study and the analysis. LL, HY, and LC contributed to collection and preparation. XY and WZ interpretated the data. YX approved the manuscript. All authors contributed to the article and approved the submitted version.

## Funding

This research was financially supported by a project of the Guangzhou University of Traditional Chinese Medicine in 2019, XK2019015; the Guan Guohua National Famous and Old Traditional Chinese Medicine Expert Inheritance Studio (Chinese Traditional Chinese Medicine human education note [2018] No. 74).

## Conflict of Interest

The authors declare that the research was conducted in the absence of any commercial or financial relationships that could be construed as a potential conflict of interest.

## Publisher's Note

All claims expressed in this article are solely those of the authors and do not necessarily represent those of their affiliated organizations, or those of the publisher, the editors and the reviewers. Any product that may be evaluated in this article, or claim that may be made by its manufacturer, is not guaranteed or endorsed by the publisher.

## Abbreviations

RDV: retinal refraction difference value
MRT: Multispectral refractive topography
AL: Axial length
LT: Lens thickness
RDV-S: RDV-Superior
RDV-I, RDV-Inferior, RDV-T: RDV-Temporal
RDV-N: RDV-Nasal
MSI: multispectral imaging technology
SE: spherical equivalent
DS: Diopter sphere
DC: Diopter cylinder.
